# Fetal Dermal Mesenchymal Stem Cell-Derived Exosomes Accelerate Cutaneous Wound Healing by Activating Notch Signaling

**DOI:** 10.1155/2019/2402916

**Published:** 2019-06-10

**Authors:** Xiao Wang, Ya Jiao, Yi Pan, Longxiao Zhang, Hongmin Gong, Yongjun Qi, Maoying Wang, Huiping Gong, Mingju Shao, Xinglei Wang, Duyin Jiang

**Affiliations:** ^1^School of Medicine, Shandong University, Jinan, Shandong 250012, China; ^2^Department of Emergency, The Second Hospital of Shandong University, Jinan, Shandong 250033, China; ^3^Department of Burns and Plastic Surgery, The Second Hospital of Shandong University, Jinan, Shandong 250033, China; ^4^Department of Pathology, The Affiliated Hospital of Qingdao University, Qingdao, Shandong 266003, China

## Abstract

Fetal dermal mesenchymal stem cells (FDMSCs), isolated from fetal skin, are serving as a novel MSC candidate with great potential in regenerative medicine. More recently, the paracrine actions, especially MSC-derived exosomes, are being focused on the vital role in MSC-based cellular therapy. This study was to evaluate the therapeutic potential of exosomes secreted by FDMSCs in normal wound healing. First, the in vivo study indicated that FDMSC exosomes could accelerate wound closure in a mouse full-thickness skin wound model. Then, we investigated the role of FDMSC-derived exosomes on adult dermal fibroblast (ADFs). The results demonstrated that FDMSC exosomes could induce the proliferation, migration, and secretion of ADFs. We discovered that after treatment of exosomes, the Notch signaling pathway was activated. Then, we found that in FDMSC exosomes, the ligands of the Notch pathway were undetectable expect for Jagged 1, and the results of Jagged 1 mimic by peptide and knockdown by siRNA suggested that Jagged 1 may lead the activation of the Notch signal in ADFs. Collectively, our findings indicated that the FDMSC exosomes may promote wound healing by activating the ADF cell motility and secretion ability via the Notch signaling pathway, providing new aspects for the therapeutic strategy of FDMSC-derived exosomes for the treatment of skin wounds.

## 1. Introduction

The skin is the largest tissue of the human body and its main function is to guard the underlying tissues. Wound healing is a complex process, and successful cutaneous wound healing needs a series of steps including inflammation, new tissue formation, and remodeling. Furthermore, skin cell migration, proliferation, differentiation, and apoptosis make great contributions to this process. These steps are tightly coordinated and well regulated to restore the multilayered structure of the skin in the normal wound-healing process [1]. Dermal fibroblasts are one of the most important cell lines involved in the normal wound-healing process [2]. The main functions of the dermal fibroblast are extracellular matrix (ECM) production, collagen synthesis, wound contraction, reepithelialization, and tissue remodeling. Once hurt, hemostasis takes place immediately. Fibroblasts, along with other cells including neutrophils, macrophages, and endothelial cells, are attracted to the wound by the blood clot. Then, fibroblasts are activated by macrophages and play a vital role in the proliferative and remodeling phase. Fibroblasts start proliferating and producing ECM proteins like collagen, hyaluronan, and fibronectin to provide a foundation of wound repair [3]. There is a paucity of pharmacological therapeutics that can accelerate wound healing of a large area burn wound and chronic, nonhealing wounds. These wounds adversely affect the life quality of the patients and put great economic pressures on the family and society. Therefore, it is important to seek an effective therapeutic method to promote wound healing [4].

Mesenchymal stem cells (MSCs) have a significant promise for regenerative medicine. Previous studies demonstrated the therapeutic potential of MSCs for tissue regeneration, including the liver, heart, bone, cartilage, neural, and skin [5–10]. Recent literature suggests that the regenerative effect of MSCs is mainly mediated through paracrine signaling to regulate host cells, instead of cell replacement [5, 11]. Fetal dermal MSCs (FDMSCs), which are derived from the dermis of accidentally aborted fetuses, exhibit advantages of high expansion potential, high differentiation properties, and low immunogenicity. As an advantageous MSC source, FDMSCs have great potential in the tissue regeneration field for their scarless wound-healing characteristic [12–14]. In our previous research, we found that FDMSCs can inhibit the bioactivity of keloid fibroblasts by a paracrine manner.

In the last decades, researchers have shown increased interest in exosomes. Exosomes are 40-100 nm small membranous vesicles secreted by most cell types. There are nuclear acids, lipids, and proteins in them, and their main function is to transfer bioactive moleculars in cell-cell communication [15, 16]. Moreover, recent studies have shown the role of exosomes in pathogenesis, tissue regeneration, diagnosis, and drug delivery [17–21]. Exosomes are released from MSCs due to paracrine signaling and transfer their cargo of proteins, RNAs, and lipids to recipient cells to regulate the cell state and behaviors. Exosomes derived from MSCs are involved in the acceleration of wound healing [20–22]. We used the promising MSC type, FDMSCs, to investigate the paracrine effect on wound healing process in vivo and in vitro, and to analyze the signal pathway associated with this process.

Notch signaling is an evolutionarily conserved pathway with numerous functions ascribed. Studies over the past decades have proved that Notch plays key roles in stem cell maintenance, development, homeostasis regulation, and cell fate decisions, and its dysfunction can contribute to a variety of diseases in humans [23]. There are 5 ligands (delta-like- (Dll-) 1, Dll-3, Dll-4, Jagged 1, and Jagged 2) in mammals, which can activate Notch signaling. Once activated, Notch receptors are cleavaged by tumor necrosis factor alpha converting enzyme (TACE) and *γ*-secretase, which results in the release of the Notch intracellular domain (NICD). Cleaved NICD can translocate into the nucleus and conjunct with a DNA-binding protein to regulate target gene expression [24, 25]. A number of studies have identified that the Notch signal plays a critical role in wound healing by regulating the proliferation and migration of endothelial cells, keratinocytes, fibroblasts, epidermal stem cells (ESCs), and other wound-healing-related cells [25–28]. Furthermore, the cell secretion ability is under the regulation of Notch signaling [29]. In this study, we hypothesized that exosomes derived from FDMSCs can promote cutaneous wound healing via the Notch signaling pathway.

## 2. Material and Method

### 2.1. Cell Culture

FDMSCs were extracted from the dorsal skin of fetal samples while adult dermal fibroblasts (ADFs) were extracted from adult skin samples of patient surgical waste. The extraction and identification steps were described in our previous study [30]. These cells were cultured in DMEM/low glucose (HyClone, USA) containing 10% fetal bovine serum (FBS, Gibco, USA) and 1% 100 U/ml Penicillin-Streptomycin (Gibco, USA).

### 2.2. Isolation and Identification of FDMSC Exosomes

The exosomes were isolated using an ExoQuick-TC kit (SBI, USA) following the instruction. In brief, approximately 80% confluent FDMSCs were washed with PBS twice and cultured for an additional 48 hours in serum-free medium (SFM) containing 1% 100 U/ml Penicillin-Streptomycin. The CM (conditioned media) was collected and centrifuged at 3,000 × g for 15 minutes to remove cells and cell debris. The supernatant was filtered using a 0.22 *μ*m filter sterilized SteritopTM (Millipore, USA), and then the supernatant was transferred to an Amicon® Ultra-15 10K Centrifugal Filter Unit (Millipore, USA) to concentrate to 1/5 volume. Appropriate volume of ExoQuick-TC was added in the supernatant in a ratio of 1 : 5 and mixed with the supernatant. After storing at 4°C overnight, the mixture was centrifuged at 1500 × g for 30 minutes to collect the exosomes. The exosomes were quantitated using the BCA Protein Assay Kit (Beyotime, China) following the manufacturer's protocol. The morphology of the exosomes was observed using a FEI Tecnai G2 Spirit transmission electron microscope (TEM, FEI, USA) after being fixed with 2% glutaraldehyde and counterstained with 4% uranyl acetate. The exosome markers, CD63, Alix, and Tsg101, were detected by Western blot using the specific antibodies. The diameter of exosomes was measured by a ZetaView Nanoparticle Tracking Microscope (Particle Metrix Inc., USA).

### 2.3. Animal Assay

Animal experiments were approved by the Ethics Committee of the Second Hospital of Shandong University. Studies were performed in 8-10-week-old BALB/c mice weighing 25 ± 5 g. Mice were anesthetized using tribromoethanol and the dorsal hair was shaved. 1 cm × 1 cm full-thickness dermal wounds were created in the skin on the back of the mouse. 200 *μ*g FDMSC-exosomes in 200 *μ*l PBS or 200 *μ*l PBS were injected subcutaneously at four sites around the wound. On days 0, 7, and 14, digital photographs of the injury site were taken. Some mice in each group were euthanized to obtain the skin tissue samples from the wound site by dissection. These samples were collected for histopathological examination by hematoxylin and eosin (H&E) and immunohistochemistry (IHC). In IHC, primary antibodies PCNA (Servicebio, China) and CK19 (Servicebio, China) were used.

### 2.4. Exosome Internalization

Exosomes were labeled with PKH26 (Sigma-Aldrich, USA) according to the manufacturer's protocol. Briefly, 5 mg of exosomes was resuspended in 0.5 ml 2 × Diluent C. PKH26 was diluted in 0.5 ml 2 × Diluent C (4 × 10^−6^ M). Immediately mix the exosomes and dye solutions to make the final concentrations of PKH26 2 × 10^−6^ M. Then, the exosome dye suspension was incubated for 3 min with periodic mixing. 1 ml 1% BSA was then added to stop the staining. The labeled exosomes were washed by centrifugation in PBS in the Amicon® Ultra-15 10K Centrifugal Filter Unit. The labeled exosomes were added to cultures of ADFs and incubated for 8 hours at 37°C. ADFs were washed thrice, and then the nuclei were stained with DAPI. Then, the cells were observed by fluorescence microscope (Olympus, USA).

### 2.5. Western Blot

Western blotting was performed following standard protocols. Western blotting was used to identify exosome markers CD63, Alix, and Tsg101. Briefly, exosomes were resuspended by PBS and loading buffer and then heated at 95°C for 5 minutes. Cell samples were lysed in RIPA lysis buffer (Beyotime, China) on ice. Then, the samples were loaded and separated in SDS-PAGE gels and transferred onto nitrocellulose membranes (Pall Life Sciences, USA). After incubating with specific antibodies, protein expression and phosphorylation were and imaged with FluorChem Q (ProteinSimple, USA). The images were quantified using ImageJ. Primary antibodies used in this study were as follows: exosome markers Alix (1 : 1000, Abcam, USA), CD63 (1 : 500, Millipore, USA), Tsg101 (1 : 1000, Abcam, USA), activated Notch1 (1 : 1000, Abcam, USA), Jagged 1 (1 : 1000, CST, USA), Hes 1 (1 : 1000, Abcam, USA), Jagged 2 (1 : 1000, CST, USA), Dll-1 (1 : 1000, CST, USA), Dll-3 (1 : 1000, CST, USA), Dll-4 (1 : 1000, CST, USA), and GAPDH (1 : 1000, ZSGB-bio, China).

### 2.6. Cell Proliferation

2 × 10^3^ fibroblasts were seeded in 96-well plates in SFM. After overnight plating, a Cell Counting Kit (CCK-) 8 (Beyotime, China) assay was performed to evaluate the cell proliferation according to the manufacturer's protocol. In brief, cells were treated with MSC exosomes (1 *μ*g/ml, 10 *μ*g/ml, and 100 *μ*g/ml) or SFM for 24 h, and each group contained three parallel holes. 20 *μ*l CCK-8 solution was added to each well and incubated for 2 hours at 37°C. The optical density of each well was measured at 450 nm using the Victor spectrophotometer (Thermo Fisher Scientific, USA).

### 2.7. Cell Migration

The migration assay was used to analyze the migration effect of FD-MSC exosomes to ADFs. 1 × 10^4^ ADFs were seeded in the upper chamber in FDMSC exosomes (1 *μ*g/ml, 10 *μ*g/ml, and 100 *μ*g/ml) or vehicle, and the bottom chambers contained culture media containing 10% FBS and 1% P/S. 24 hours later, cells were fixed with 4% paraformaldehyde and stained with 0.1% crystal violet, and then the upper surface cells were removed with a cotton swab. The cells of the lower membrane surface were counted under a microscope (Nikon, Japan) at ×100 magnification, and 5 random fields were selected.

### 2.8. Quantitative Real-Time PCR

The primers were synthesized by BGI (China), and the sequences are listed in Table 1. Total RNA was isolated from ADFs and mouse-excised skin wounds using a TRIzol reagent (Invitrogen, USA) according to the manufacturer's instructions. cDNA was synthesized using a HiScript II Q RT SuperMix for qPCR (Vazyme Biotech, China), and real-time PCR was performed using a SYBR Green Master Mix (TAKARA, China) reagent. GAPDH was used as the reference gene for calculations. The *ΔΔ*Ct method was used to analyze the real-time PCR data.

### 2.9. DAPT Treatment

*γ*-Secretase inhibitor DAPT was purchased from Sigma-Aldrich. In an inhibiting assay, ADFs were incubated with SFM, 100 *μ*g/ml FDMSC exosomes, or 10 *μ*M DAPT + 100 *μ*g/ml FDMSC exosomes for 24 hours. Then, the cell proliferation and migration were evaluated by CCK-8 and Transwell assays.

### 2.10. Jagged 1 Peptide Treatment

Jagged 1 peptide (CDDYYYGFGCNKFCRPR) with Notch agonist activity and scrambled control (SC) peptide (RCGPDCFDNYGRYKYCF) was synthesized at the Qiangyao Biological Technology Company (China) [31]. Peptide stock solutions (10 mM) were prepared in sterile distilled water and diluted to 15 *μ*M in culture medium before use.

### 2.11. siRNA Knockdown

FDMSCs were transfected with Jagged 1 siRNA and control RNA (RiboBio, China). A Lipofectamine™ RNAiMAX Transfection Reagent (Thermo Fisher Scientific, USA) was used according to the manufacturer's instructions. 10 hours after transfection, ADFs were washed with PBS twice and cultured with SFM. 48 hours later, CM was collected from Jagged 1 siRNA or control siRNA-transfected FDMSCs to isolate Jagged 1 siRNA exosomes and control siRNA exosomes separately.

### 2.12. Statistical Analysis

Statistical analyses were performed using GraphPad Prism 5. Three or more independent experiments were performed for each result, and the mean and SD were calculated. One-way ANOVA or Student's *t*-test was used to detect statistically significant differences. A *P* value < 0.05 was considered as statistically significant.

## 3. Results

### 3.1. Characterization of FDMSC Exosomes

**Figure 1:**
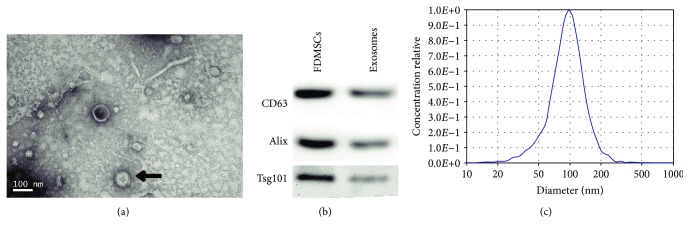
Characterization of FDMSC exosomes. (a) Morphology of FDMSC exosomes under TEM (black arrow). Scale Bar = 100 nm. (b) Western blotting analysis of exosomal CD63, Alix, and Tsg101 protein in FDMSC exosomes. (c) Diameter analysis of FDMSC exosomes.

FDMSCs were successfully isolated from fetus dorsal skin and identified by flow cytometry analysis and differentiation potential analysis in our previous study [30]. FDMSC exosomes were isolated and then analyzed by TEM and Western blotting. We used TEM to analyze the size, shape, and morphology of exosomes, and the result clearly revealed that FDMSC exosomes have a size range of about 100 nm with an appearance of cup-shaped or round-shaped morphology (Figure 1(a), black arrow). The result showed the presence of exosome marker proteins, CD63, Tsg101, and Alix, in exosome lysates (Figure 1(b)). Then, we measured the size of the exosomes, and the results showed that the diameter of exosomes was about 100 nm (Figure 1(c)).

### 3.2. FDMSC Exosomes Promote Cutaneous Wound Healing In Vivo

**Figure 2:**
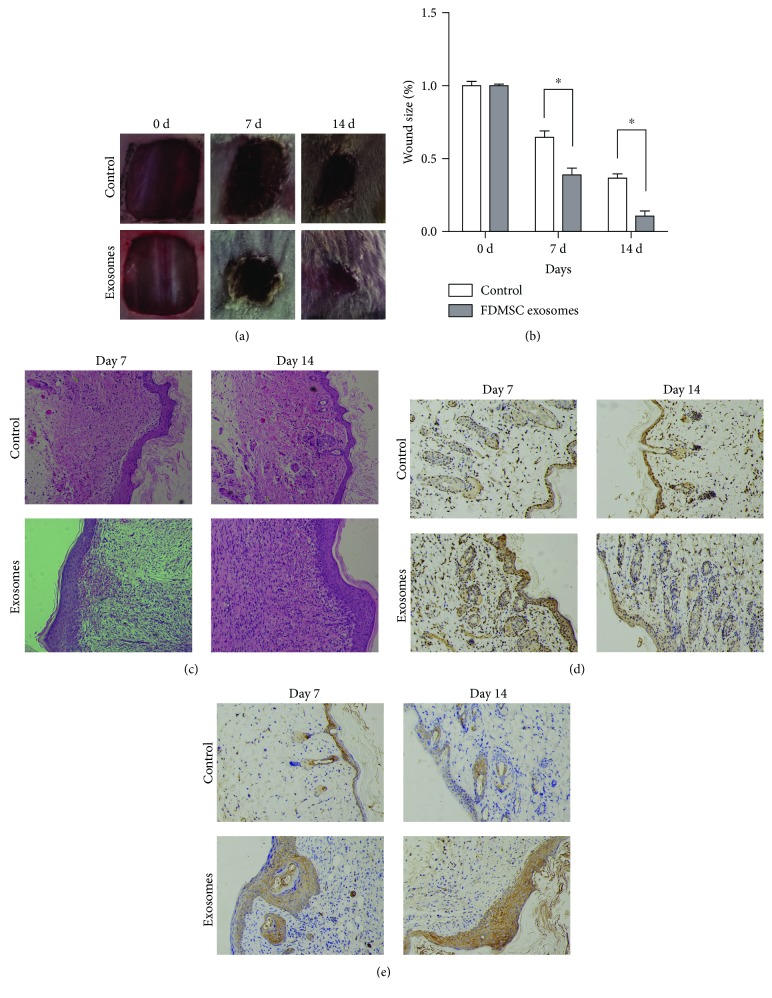
FDMSC exosomes accelerate cutaneous wound healing in vivo. (a) Representative photos of mouse dorsal full-thickness wound healing. (b) Quantitative analysis of wound size (*n* = 5 − 7 per group). (c) Representative H&E stain images of the wound at 1 week and 2 weeks after treatment (×100 magnification). (d) Representative images of IHC of PCNA in each group (×200 magnification). (e) Representative images of IHC of CK19 in each group (×200 magnification). ^∗^*P* < 0.05.

We established a mouse full-thickness dermal wound injury model to investigate the roles of FDMSC exosomes in wound healing. In the exosome-treated group, the wounds healed more rapidly than those in the control group (Figures 2(a) and 2(b)). H&E results indicated that in the exosome-treated group, there are more cells in ECM and the ECM proteins are more regular and denser, with a thicker layer of collagen than that in the control group on 7 days and 14 days posttreatment (Figure 2(c)). Furthermore, in FDMSC exosome-treated wounds, there were more cells with a higher proliferative rate in the wound area evaluated by PCNA IHC results (Figure 2(d)). The IHC result of CK19 illustrated that the reepithelialization in the exosome-treated group was accelerated and the regenerative epidermis is thicker than that in the control group (Figure 2(e)). In summary, our in vivo results indicated that FDMSC exosomes can accelerate cutaneous wound healing by promoting cell proliferation, ECM deposition, and reepithelialization in the wound area.

### 3.3. FDMSC Exosomes Enhance Proliferation, Migration, and Secretion of ADFs

**Figure 3:**
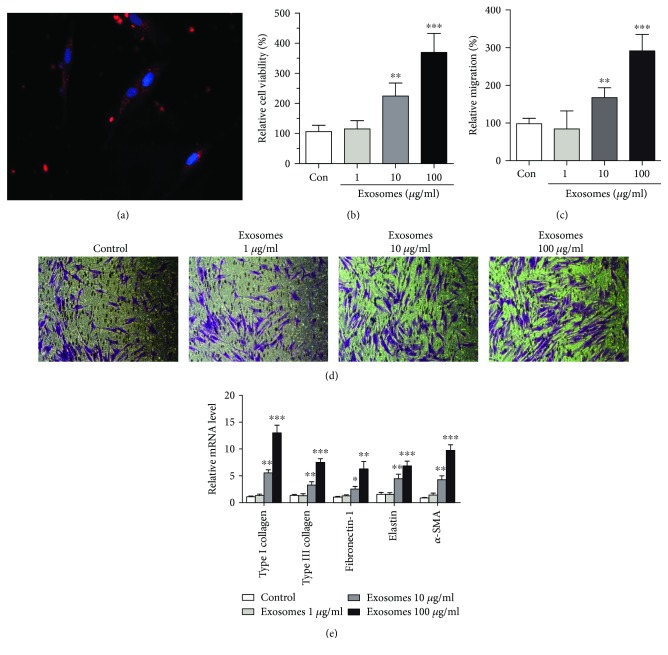
FDMSC exosomes regulate proliferation, migration, and secretion of ADFs. (a) The internalization of exosomes by ADFs. (b) FDMSC exosomes can significantly stimulate ADF proliferation in a dose-dependent manner. (c, d) Transwell assay of the migration of ADFs in different exosome-treatment groups. (c) Cell migration is expressed as a percentage of control. (d) Representative images of the migration (×100 magnification). Cells were counted for at least five random microscope fields. Results are shown as mean ± SD from three independent experiments. (e) FDMSC exosomes promote ECM protein secretion of ADFs. Real-time PCR analysis of the mRNA levels of Type I and III collagen, elastin fibronectin, and *α*-SMA genes (normalized to GAPDH) in ADFs of different groups. Results are shown as mean ± SD of three independent experiments. Two-tailed Student's *t*-test was used. ^∗∗∗^*P* < 0.001, ^∗∗^*P* < 0.01, or ^∗^*P* < 0.05.

The main functions of ADFs are to synthesize, secrete, and deposit collagen and elastic fibers of the ECM. Therefore, the proliferation, migration, and protein synthesis abilities of ADFs are vital factors in wound healing. To explore the mechanisms for FDMSC exosome-induced repair, we treated ADFs with FDMSC exosomes. FDMSC exosomes (red) were found to be internalized by the ADFs (Figure 3(a)). To determine the effect of FDMSC exosomes on ADF growth and mobility, CCK-8 and Transwell assays were performed. The results showed that cell proliferation ability of ADFs was significantly improved after being treated with exosomes in a dose-dependent manner (Figure 3(b)). Compared to the control group, the migratory capabilities of ADFs were also significantly improved in the presence of exosomes (Figures 3(c) and 3(d)). These results demonstrated that exosomes significantly enhanced the proliferation and migration of ADFs in a concentration-dependent manner. Fibroblasts, due to their abilities of synthesis and secretion of ECM proteins, play a significant role during repair of skin wounds. These proteins, to a certain extent, determine the speed and quality of wound healing. Here, we analyzed the mRNA expressions of ECM proteins and wound-healing-related proteins (Type I and III collagen, fibronectin, elastin, and *α*-SMA) of ADFs by real-time PCR after being treated with FDMSC exosomes. We found that in ADFs incubated with exosomes (1 *μ*g/ml, 10 *μ*g/ml, and 100 *μ*g/ml) for 48 hours, Type I and III collagen, elastin, and fibronectin mRNA production was increased in a dose-dependent manner (Figure 3(e)). The results suggested that the FDMSC exosomes can promote the ECM secretion of ADFs.

### 3.4. FDMSC Exosomes Activate the Notch Signaling Pathway

**Figure 4:**
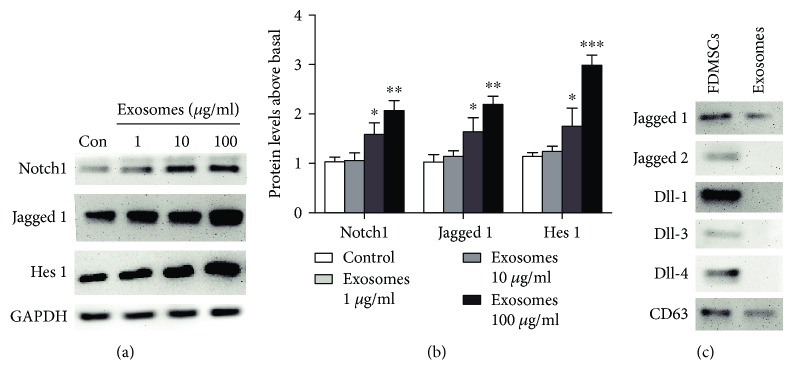
FDMSC exosomes activate the Notch signal pathway. (a, b) Western blot analysis of key Notch signaling-related protein levels in ADFs treated with SFM or exosomes. GAPDH served as a loading control. Notch1, Jagged 1, and Hes 1 were upregulated in ADF by exosome treatment. (c) Western blot analysis of Notch ligands in FDMSC exosomes. Jagged 1 is the only Notch ligand expressed in FDMSC exosomes while Jagged 2, Dll-1, Dll-3, and Dll-4 were undetectable. CD63 was used as a loading control of exosomes. Two-tailed Student's *t*-test was used. ^∗∗∗^*P* < 0.001, ^∗∗^*P* < 0.01, or ^∗^*P* < 0.05.

Recently, researchers have shown the importance of the Notch signal in skin development and tissue regeneration. Therefore, we hypothesized that Notch signaling might be involved in the exosome-mediated wound-healing process. To investigate the underlying mechanism of the effect of FDMSC exosomes on ADFs, the expression level of Notch1, Jagged 1, components of the Notch signaling pathway, and hairy and enhancer of split-1 (Hes 1), a Notch target gene, were analyzed by Western blot. The results showed the increased expression of active Notch1, Jagged 1, and Hes 1, which illustrated the activation of Notch signaling in the presence of FDMSC exosomes (Figures 4(a) and 4(b)). To find how the Notch signaling was activated, we detected the Notch ligands in exosomes and found that Jagged 1 was packaged into MSC exosomes while the others were undetectable by Western blot (Figure 4(c)).

### 3.5. DAPT Can Partly Block the Promoting Effect of FDMSC Exosomes of ADF Proliferation and Migration

**Figure 5:**
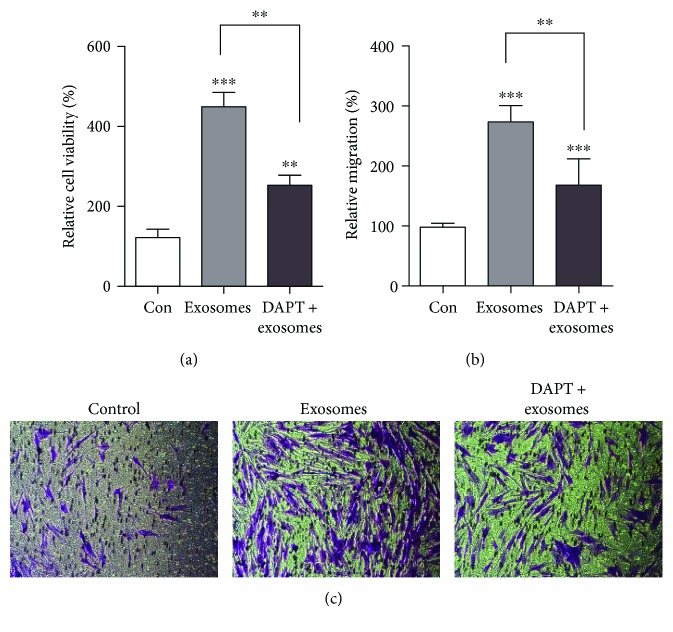
Activated Notch signaling by FDMSC exosomes can be partly blocked by DAPT. (a) DAPT can partly block the proliferation of ADFs. (b, c) DAPT can partly block the migration of ADFs. (b) Cell migration is expressed as a percentage of control. (c) Representative images of the migration (×100 magnification). ^∗∗∗^*P* < 0.001 or ^∗∗^*P* < 0.01.

To determine whether exosomes can promote ADF proliferation and migration in a Notch-dependent manner, we treated ADFs with DAPT, the *γ*-secretase inhibitor, to block Notch receptor cleavage at the cell surface. ADFs were treated with SFM, 100 *μ*g/ml exosomes or DAPT (10 *μ*M) + exosomes (100 *μ*g/ml). We found that DAPT partly abolished the positive regulating effect in cell proliferation (Figure 5(a)) and migration (Figures 5(b) and 5(c)) of FDMSC exosomes on ADFs. These results indicate that FDMSC exosomes can activate the wound-healing capacity of ADFs via the Notch signaling pathway, and these effects can also be inhibited when DAPT was used, illustrating the role of the Notch pathway in wound healing.

### 3.6. Jagged 1 in FDMSC Exosomes Promote the Wound-Healing Capacity of ADFs

**Figure 6:**
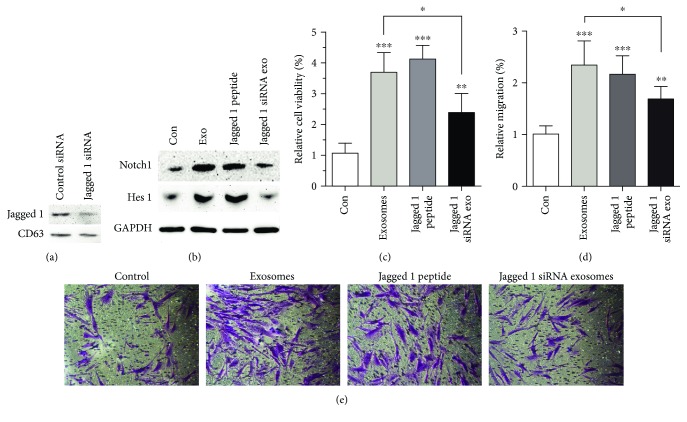
Jagged 1 in FDMSC exosomes can promote the wound-healing capacity of ADFs. (a) The expression of Jagged 1 in FDMSC exosomes was knocked down by siRNA. (b) Western blot analysis of Notch1 and Hes 1 protein levels in ADFs treated with SFM, FDMSC exosomes, Jagged 1 peptide, or Jagged 1 siRNA exosomes. GAPDH served as a loading control. Notch1 and Hes 1 were upregulated in the exosome and Jagged 1 peptide treatment groups, while in Jagged 1 siRNA exosome treatment group, the activating effect of FDMSC exosomes on the Notch signal was abolished after Jagged 1 knockdown. (c) The proliferation of ADFs. (d, e) The migration of ADFs. (d) Cell migration is expressed as a percentage of control. (e) Representative images of the migration (×100 magnification). ^∗∗∗^*P* < 0.001, ^∗∗^*P* < 0.01, or ^∗^*P* < 0.05.

To further investigate the functional role of Jagged 1 expressed in exosomes in wounding, we used the Jagged 1 peptide to mimic Jagged 1 in activating the Notch signal and knockdown Jagged 1 expression in FDMSCs by siRNA. The expression of Jagged 1 in FDMSC exosomes was reduced after siRNA knockdown (Figure 6(a)). ADFs were incubated with SFM, 100 *μ*g/ml FDMSC exosomes, 15 *μ*M Jagged 1 peptide, or 100 *μ*g/ml Jagged 1 knockdown exosomes for 24 hours. We found that in the FDMSC exosome and Jagged 1 peptide treatment groups, the Notch pathway was activated, and the proliferation and migration ability of ADFs was increased, while depletion of Jagged 1 in FDMSC exosomes by siRNA blocked the activation of Notch signaling and blocked the promoting ability of FDMSC exosomes on the proliferation and migration of ADFs (Figures 6(b)–6(e)). These results indicated that Jagged 1 in FDMSC exosomes can activate the wound-healing capacity of ADFs via the Notch signaling pathway.

## 4. Discussion

Wound healing is an integrated and coordinated process of different cells functionally relevant to skin tissue repair, alone with the microenvironment around them. There are a large number of published studies that describe the treatment methods for the management of cutaneous wound healing; however, the questions and difficulties are still remaining in this field. Especially for nonhealing and chronic wounds, effective therapeutic approaches need to be further explored to deal with this prevalent and costly public health issue. Thus, it is urgent to find an effective approach to prompt wound healing [32].

In the last decades, researches and clinical trials of MSC applications in tissue regeneration have made great progress. Studies focused on MSC transplantation suggested that instead of direct cell differentiation and replacement, MSCs play regulation and stimulation roles via paracrine signaling by releasing factors that promote angiogenesis, immunomodulation, and recruitment of different cells [5, 9, 33]. Literature has proved the positive effect of MSC CM on tissue regeneration [5, 34, 35]. Growth factors, cytokines, immunomodulatory proteins, and other biologically active proteins are the major components of CM. Besides, the discovery of exosomes helps us gain a better understanding of the underlying mechanism of the multiple effects of MSCs throughout the body [36–38]. Exosomes can mediate the cell-cell communication by transferring RNAs, proteins, and lipids to recipient cells and modifying their bioactivity state [15]. Nowadays, exosomes are considered as novel therapeutic tools and diagnostic markers [17, 39, 40].

MSC exosomes can exhibit repair effect, consistently with the MSCs, on the injured tissues through modifying recipient cell gene expression, protein production, and status, as well as activating regeneration-associated pathways including Wnt/*β*-catenin, AKT, ERK, and STAT3 [41–43]. Recently, investigators have examined the regenerative effects of exosomes derived from MSCs on tissues of the lung, heart, kidney, liver, brain, and so on. Therefore, exosomes derived from MSC exosomes may become potential therapeutic agents in cell-free tissue regeneration therapy. According to advanced research in wound healing, MSC exosomes can increase the proliferation and migration of skin cells and inhibit their apoptosis.

Fetal MSCs are a new potential source of MSCs. The dorsal skin of aborted fetuses, which is considered as clinic discards, is an alternative abundant source of MSCs, and the clinic significance needs to be further explored. Compared with adult MSCs, fetal MSCs exhibit low immunogenicity, higher proliferation, and differentiation potential. FDMSCs are derived from accidental aborted fetuses, and they are thought as the main functional cells involved in scarless wound healing [12]. Furthermore, owing to the histological origin of FDMSCs, they may deserve unique properties on skin regeneration. In summary, FDMSCs are better candidates than adult MSCs in wound healing.

Fibroblasts, as the important target of exosomes in wound healing, are the major cell type to synthesize, secrete, and deposit collagen and elastic fibers of the ECM [2]. Recently, there has been renewed interest in the different fibroblast lineages [24, 44]. Researchers found that fibroblasts isolated from different dermal sources exhibit diverse functions, and the underlying mechanism needs to be explored further. Fibroblasts from diabetic patients showed impaired function in wound healing with reduced migration response and growth factor expression [45, 46]. In summary, the proliferation, migration, and protein synthesis abilities of dermal fibroblasts are vital for wound repair. Activation of fibroblasts in the early phase of wound healing can accelerate the wound closure and matrix protein production, providing a foundation for wound repair.

In our study, results suggested that FDMSC exosomes have an enhancing effect on ADF cell growth and migration. Further analysis by real-time PCR showed significantly elevated ECM protein levels compared to those of the control group, indicating that FDMSCs can promote ECM protein synthesis. The upregulation of Notch1, Jagged 1, and Hes 1 exhibited the activating effect of FDMSC exosomes on Notch signaling. Furthermore, Western blot analysis of exosome components showed Jagged 1 was the only ligand that can be detected, and the inhibition of Notch signaling by DAPT significantly decreased the proliferation and migration of ADFs. In contrast, ADFs treated with FDMSC exosomes and Jagged peptide showed significantly enhanced proliferation and migration, and knockdown of Jagged 1 in exosomes abolished the promoting effect. These results emphasizing that the Notch pathway is a mediator of exosome communication in regulating wound repair and Jagged 1 in exosomes play a vital role.

As one of the important Notch ligands, Jagged 1 can regulate maturation of the human epidermis by activating Notch signaling [31]. In addition, Jagged 1 is present in exosomes from different kinds of cells and is biologically active, but the role of Jagged 1 in exosomes in wound healing is largely unknown [47–49]. In this study, we found that Jagged 1 is sorted in FDMSC exosomes to regulate the Notch signal pathway activity in ADFs. However, the quantity of Jagged 1 in FDMSC exosomes is variable and unstable because the biogenesis of exosomes is largely depending on cell types, cell functions, and physiological statuses. Due to the complexity of FDMSC exosomes, the important components of the exosomal cargo and other factors which can activate Notch signaling and the mechanism are still studying. Further research is needed to elucidate a detailed molecular mechanism of the sorting process and biological functions of Jagged 1 and the exact mechanism of FDMSC exosomes in wound healing and to develop new therapeutic strategies for nonhealing and chronic wounds.

In conclusion, we successfully obtained FDMSC exosomes and investigated their role on cutaneous wound healing. Our results demonstrated that FDMSC exosomes can exert promoting effect on the proliferation, migration, and protein synthesis abilities of ADFs via Notch signal activation.

## 5. Conclusion

The results demonstrated that FDMSC exosomes could accelerate cutaneous wound healing in vivo and promote the wound-healing capacities of ADFs by activating the Notch signal pathway in vitro. Our findings provided new aspects for the therapeutic strategy of FDMSC-derived exosomes for the treatment of skin wounds.

## Figures and Tables

**Table 1:** Primer sequences for quantitative real-time PCR.

Gene name	Forward	Reverse
Type I collagen	5′-CGGCGAGAGCATGACCGATGG-3′	5′-TCCATGTAGGCCACGCTGTTC-3′
Type III collagen	5′-ACAAAGAGGAGAACCTGGACC-3′	5′-GGAGGACCCCGGGCTCCCATC-3′
Fibronectin-1	5′-AATAGCCCTGTCCAGGAGTTCA-3′	5′-GTAATTAATGGAAATTGGCTTGC-3′
Elastin	5′-GAGGCAAACCTCTTAAGCC-3′	5′-AGCCCAGCGCCAGCCTTAGCAGCT-3′
*α*-SMA	5′-CATCACCAACTGGGACGACA-3′	5′-TCCGTTAGCAAGGTCGGATG-3′
GAPDH	5′-GCACCGTCAAGGCTGAGAAC-3′	5′-TGGTGAAGACGCCAGTGGA-3′

## Data Availability

The data used to support the findings of this study are available from the corresponding author upon request.

## Abbreviations

*α*-SMA: *α*-Smooth muscle actin
ADFs: Adult dermal fibroblasts
CM: Conditioned media
ECM: Extracellular matrix
FDMSCs: Fetal dermal mesenchymal stem cells
H&E: Hematoxylin and eosin
Hes 1: Hairy and enhancer of split-1
IHC: Immunohistochemistry
NICD: Notch intracellular domain
SFM: Serum-free medium
siRNA: Small interfering RNA
TACE: Tumor necrosis factor alpha converting enzyme.
